# Structure of the yeast Swi/Snf complex in a nucleosome free state

**DOI:** 10.1038/s41467-020-17229-x

**Published:** 2020-07-07

**Authors:** Chengcheng Wang, Zhouyan Guo, Xiechao Zhan, Fenghua Yang, Mingxuan Wu, Xiaofeng Zhang

**Affiliations:** 1 Key Laboratory of Structural Biology of Zhejiang Province, Institute of Biology, Westlake Institute for Advanced Study, 18 Shilongshan Road, 310024 Hangzhou, Zhejiang Province China; 2 School of Life Sciences, Westlake University, 18 Shilongshan Road, 310024 Hangzhou, Zhejiang Province China; 3 College of Life Sciences, Zhejiang University, 310058 Hangzhou, China; 4 Beijing Advanced Innovation Center for Structural Biology, Tsinghua-Peking Joint Center for Life Sciences, School of Life Sciences, Tsinghua University, 100084 Beijing, China; 5 School of Science, Westlake University, 18 Shilongshan Road, 310024 Hangzhou, Zhejiang Province China; 6 Institute of Natural Sciences, Westlake Institute for Advanced Study, 18 Shilongshan Road, 310024 Hangzhou, Zhejiang Province China

**Keywords:** Biochemistry, Cryoelectron microscopy, Molecular biology

## Abstract

SWI/SNF remodelers play a key role in regulating chromatin architecture and gene expression. Here, we report the cryo-EM structure of the *Saccharomyces cerevisiae* Swi/Snf complex in a nucleosome-free state. The structure consists of a stable triangular base module and a flexible Arp module. The conserved subunits Swi1 and Swi3 form the backbone of the complex and closely interact with other components. Snf6, which is specific for yeast Swi/Snf complex, stabilizes the binding of the ATPase-containing subunit Snf2 to the base module. Comparison of the yeast Swi/Snf and RSC complexes reveals conserved structural features that govern the assembly and function of these two subfamilies of chromatin remodelers. Our findings complement those from recent structures of the yeast and human chromatin remodelers and provide further insights into the assembly and function of the SWI/SNF remodelers.

## Abstract (web summary)

SWI/SNF remodelers alter histone-DNA interactions and are important for regulating chromatin architecture and gene expression. Here, the authors present the cryo-EM structure of a nucleosome-free Swi/Snf complex from *Saccharomyces cerevisiae* and discuss the assembly and function of SWI/SNF remodelers.

## Introduction

SWI/SNF remodelers, which are highly conserved from yeast to humans, alter histone−DNA interactions and consequently play critical roles in the regulation of chromatin architecture and gene expression. Yeast SWI/SNF mutants exhibit many mutant phenotypes, including poor growth, the inability to use particular carbon sources, and defects in sporulation^1,2^. In humans, mutations of the SWI/SNF subunits are frequently found in 20% of cancers^3^ and obviously play a major role in cancer development and progression^4^.

In most eukaryotes, there are two subfamilies of SWI/SNF remodelers, Swi/Snf or RSC in yeast, and Brg1/Brm-associated factor (BAF) or polybromo-associated BAF (PBAF) in mammals. The *Saccharomyces cerevisiae* Swi/Snf complex, which was the first chromatin remodeler discovered, consists of 12 proteins with a combined molecular weight of ~1 megadaltons (MDa)^5–7^. The subsequently identified SWI/SNF complexes contain both conserved proteins and unique subunits to help each complex specialize and carry out different functions^3,8,9^.

Although the components of the *S. cerevisiae* Swi/Snf complex were identified in the 1990s^10–14^, how these subunits contribute to the stability and function of the complex is poorly understood. Early studies demonstrated that at least four subunits of the Swi/Snf complex (Swi1, Swi3, Snf5, and Snf6) are required for assembly into a high-molecular weight complex with Snf2^10,15^. In contrast, Swp82, Taf14, and Snf11 do not seem to be essential for Swi/Snf complex function in vivo^16,17^.

Recently, structures of the yeast RSC and the human BAF complexes bound to nucleosomes at moderate resolution have been reported^18–20^, revealing their respective mechanisms of subunit organization and nucleosome recognition. Here, we report the cryo-electron microscopy (cryo-EM) structure of the *S. cerevisiae* Swi/Snf complex in a nucleosome-free state. Using a combination of de novo modeling, homology building, and structure docking, we have generated a complete atomic model of the yeast Swi/Snf complex. Although the overall structure is similar to that of other SWI/SNF complexes, it still reveals a set of striking features that advance mechanistic understanding of complex organization.

## Results

### Architecture of the yeast Swi/Snf complex in nucleosome-free state

**Fig. 1:**
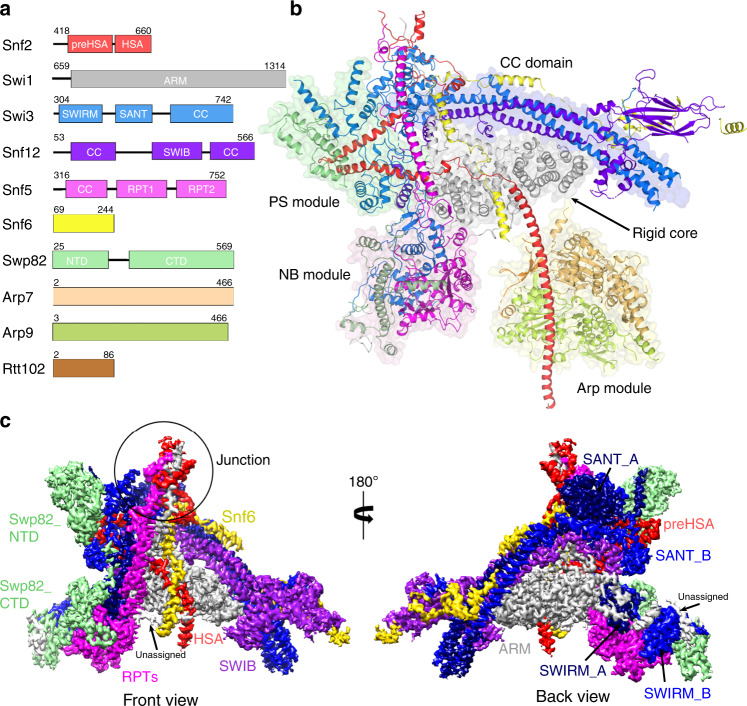
Overall structure of the yeast Swi/Snf complex in the nucleosome-free state. **a** Domain organization of all subunits that are included in the final model. **b** Cartoon model of the Swi/Snf complex with the individual subunits colored. The rigid core, PS module, NB module, CC domain, and Arp module are colored in gray, green, pink, and orange, respectively. **c** Front (above) and back (below) views of the cryo-EM composite map of the base module locally refined to 2.9 Å. The chain A of Swi3 is colored in deep blue while the chain B is colored in bright blue.

The intact 12-subunits Swi/Snf complex was endogenously purified from *S. cerevisiae* by employing a C-terminal Flag tag on Snf6 (Supplementary Fig. 1). The isolated complexes exhibited excellent solution behavior as judged by gel filtration. Cryo-EM analysis of 7746 micrographs gave a reconstruction of the Swi/Snf complex at an average resolution of 4.1 Å, with the local resolution of the core reaching 2.9 Å (Supplementary Figs. 2–4).The high-quality EM map allowed atomic modeling covering ten components including two copies of Swi3 (Fig. 1a, b). Taf14 and Snf11, the two other subunits present in the purified sample (Supplementary Fig. 1c, d), could not be identified in the final map, likely due to their highly mobile nature. The overall structure can be divided into five main lobes, corresponding to the rigid core, the nucleosome binding (NB) module, the preHSA stabilization (PS) module, the coiled coil (CC) domain, and the Arp module, respectively (Fig. 1b). The first four lobes form a stable triangular base module (Fig. 1c) that consists of Swi1, Swi3, Snf5, Snf12, Snf6, Swp82, and preHSA domain of Snf2. The Arp module (Snf2-HSA, Arp7, Arp9, and Rtt102) hangs on one edge of the triangle and is loosely connected to the base. The flexibility of the Arp module and the absence of the ATPase domain of Snf2 in the structure could be caused by the absence of the nucleosome.

The rigid core is located in the center of the whole complex and stacked against the armadillo (ARM) repeats of Swi1. The NB module, which is supposed to bind the nucleosome^20,21^, is positioned in one corner of the triangle. In the NB module, the RPT domains of Snf5 are highly conserved from yeast to mammals and interact with the SWIRM domains of Swi3A and Swi3B by electrostatic interaction (Supplementary Fig. 5a−c). In addition, Swp82_CTD stitches the RPT and SWIRM domains together (Supplementary Fig. 5b, d). The PS module is above the NB module and forms another corner of the triangle, where Swp82_NTD and SANT_B together stabilize the preHSA helices (residues 418–557) of Snf2. The CC domain is formed by the α helices from Swi3 and Snf12. Together, they build the outer frame of the triangle. Snf2, Swi1, Snf6, Swi3, and Snf5 merge at a multiway junction, maintaining complex stability in the process of remodeling.

By comparing with the yeast RSC and human BAF complexes, we find that yeast Swi/Snf complex reveals not only conserved structural features but also unique properties; this will be discussed in detail in the following sections.

### Swi1 serves as a rigid core and forms the backbone with Swi3

**Fig. 2:**
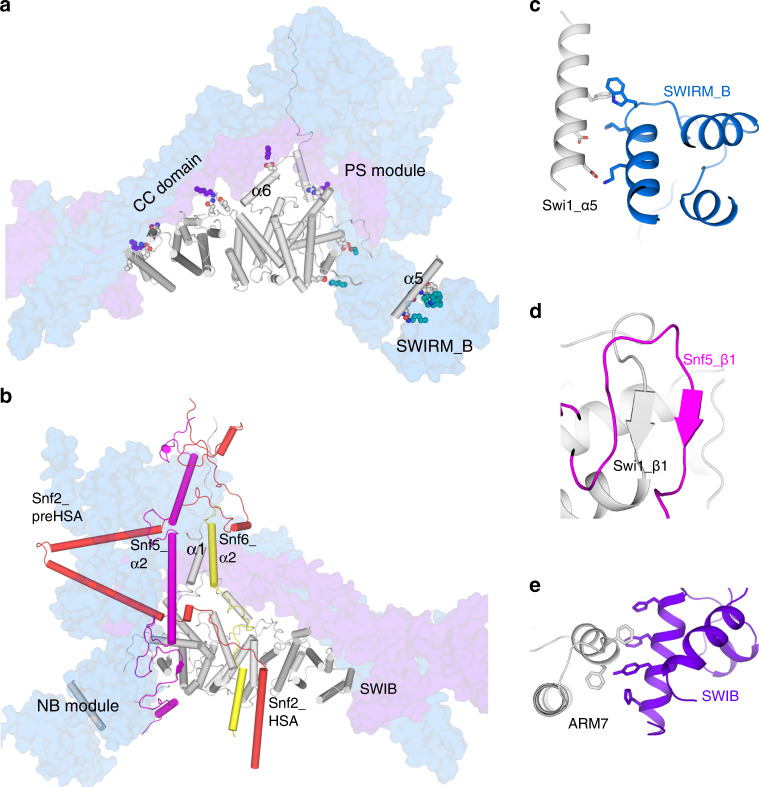
Swi1 serves as a rigid core. **a** Interactions between Swi1, Snf12, and Swi3A/B. The residues involved are shown with sphere model. Swi1 is shown as cartoon model colored in gray. Swi3A/B and Snf12 are shown as surface models colored in blue and purple blue, respectively. **b** Interactions between Swi1, Snf2, Snf5, and Snf6. Snf2, Snf5, and Snf6 are shown as cartoon models colored in red, magenta, and yellow, respectively. **c** The α5 interacts with SWIRM_B domain. **d** The β sheet formed by Swi1_β1 and Snf5_β1. **e** The interface between ARM7 from Swi1 and SWIB domain from Snf12.

The C-terminus of Swi1, consisting of seven ARM repeats and four additional α-helices (α1, 5, 6, and 14) (Supplementary Fig. 6a), interacts with the following proteins: Snf2, Snf5, Snf6, Snf12, and Swi3. The long side of the ARM repeats connects with the CC domain through hydrophobic bonds, whereas the short side binds PS module and NB module (Fig. 2a, b). Notably, an extended α helix (Swi_α5) contacts the NB module by interacting with SWIRM_B domain of Swi3 (Fig. 2a, c and Supplementary Fig. 4d). The β1 strand of Swi1 is parallel to the β1 strand of Snf5 (Fig. 2b, d and Supplementary Fig. 4e). The seventh ARM repeat (ARM7) is stacked against aromatic residues from the SWIB domain of Snf12 (Fig. 2b, e and Supplementary Fig. 4f). Thus, Swi1 serves as the rigid core of the Swi/Snf complex and plays a key role in stabilizing the base module, consistent with previous genetic and biochemical observations^22–24^.

Adjacent to the N-terminus of ARM repeats of Swi1, an α helix (Swi1_α1) is parallel to Snf6_α2 (residues 103–122) and Snf5_α2 (residues 371–400), forming a clamp-like structure that sandwiches the preHSA loop (residues 558–575) (Fig. 2b). Intriguingly, the structural features of Swi1_α1 are similar to those of Baf250A_α1 ^20^ (Supplementary Fig. 6b). Moreover, it was reported that the absence of Baf250A (the human homolog of Swi1) disrupted the interaction of Brg1 (the human homolog of Snf2) with the base module^25^. Both these structural features as well as biochemical data suggest that Swi1 forms a rigid core to not only stabilize the base module but also bridge Snf2 and the base module.

In the periphery of the core, the Swi3 dimer, consisting of coiled-coils, SANT, and SWIRM domains, forms an “L” shape and enwraps Swi1 (Fig. 2a, b). Together, they form the backbone of the whole complex, which is consistent with structures of the yeast RSC and human BAF complexes (Supplementary Fig. 7). However, Swi1-Swi3 and Baf250A-Baf170 are in the same plane in stereoscopic space, whereas the SWIRM_SANT domains of Rsc8 are rotated ~65° from the plane formed by the CC domain and ARM domain (Supplementary Fig. 7).

### Snf6 anchors the preHSA loop of Snf2 on the surface of the base module

**Fig. 3:**
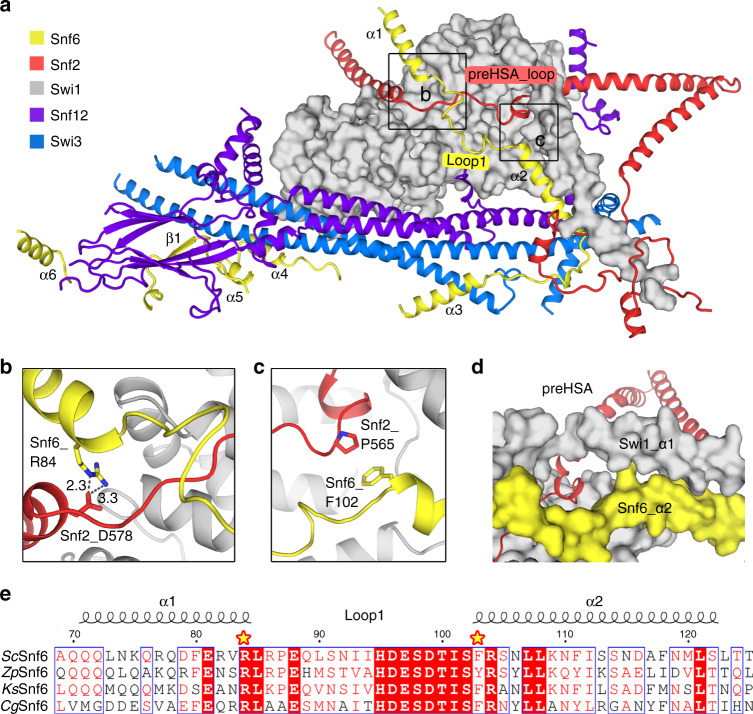
Snf6 locks the preHSA loop above the base module. **a** Shown here is the conformation of Snf6 and the surrounding proteins. Snf6 adopts an extended conformation and displays an “L” shape, with α1-2 and loop 1 (residues 85–102) stretching along the surface of Swi1 and the following sequence (α3-6 and β1) turned approximately 90° to adhere to the periphery of the CC domain. The protein elements are color coded and tabulated on the left. **b**, **c** Close-up views of the interactions between Snf6 and Snf2. **d** Snf6_Nα2 and Swi1_α1 form a “clamp-like” shape. **e** Sequence alignment of Snf6_loop 1 from different yeast species. *Sc* *Saccharomyces cerevisiae*, *Zp* *Zygosaccharomyces parabailii,* *Ks* *Kazachstania saulgeensis*, *Cg* *Candida glabrata*.

The structure of Snf6 adopts an extended conformation and displays an “L” shape, with the α1-2 and loop 1 (residues 85–102) stretching along the surface of Swi1 and the following sequences (α3-6 and β1) turned approximately 90° to adhere to the periphery of the CC domain (Fig. 3a).

Loop 1 of Snf6, which is highly conserved among *Hemiascomycetes* (Fig. 3e), is intertwined with the preHSA loop of Snf2. Arg84, the N-terminal residue of Snf6_loop 1, interacts with Asp578 from the HSA helix through hydrogen bonds (Fig. 3b), while the C-terminal residue Phe80 forms Π stacking with the residue Pro565 from Snf2 (Fig. 3c). Notably, Snf6_α2 is parallel to Swi1_α1 (Fig. 3d), clamping the preHSA domain of Snf2 to the surface of the “Swi1-Swi3” backbone.

According to previous reports, Snf6 is required to maintain the structural integrity of Swi/Snf and the loss of Snf6 impaired the association of the base module with the Arp module^10,16,21,26^. Though Snf6 is specific to certain fungi and has no homolog in mammals^27^, its structure and function are similar to those of human Baf57 (Supplementary Fig. 8a), whose deletion also results in partial dissociation of the complex^22,28^. Notably, there is no homolog of Snf6 or BAF57 in RSC complex, but the preHSA loop of Sth1 is stabilized by several unique components of the RSC complex (Supplementary Fig. 8b).

### The SANT_B domain plays central roles in stabilizing the preHSA domain

**Fig. 4:**
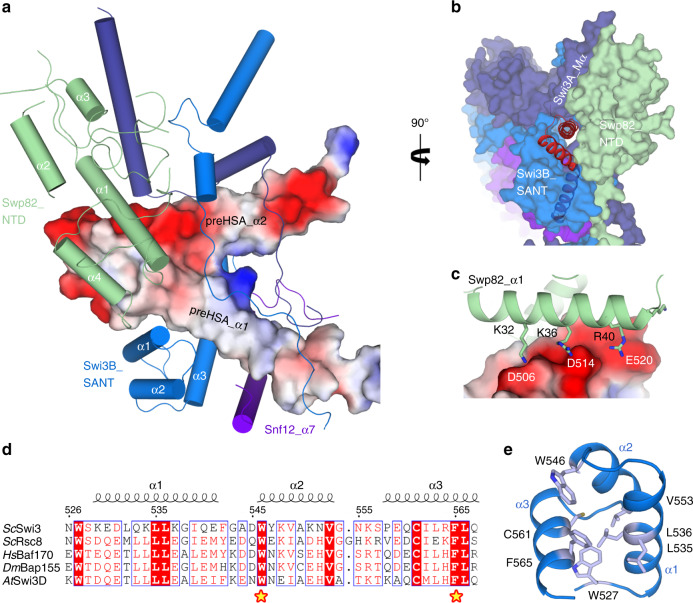
Stabilization of preHSA domain. **a** Front view of the PS module. The preHSA domain is shown as an electrostatic potential map (blue and red colors represent positive and negative charges, respectively), and the surrounding protein elements are shown in cartoon representation. **b** Side view of the PS module. **c** Close-up view of the interface between Swp82_α1 and preHSA. Key residues are labeled. **d** Sequence alignment of SANT domains of SWI/SNF complexes from different species. *Sc* *Saccharomyces cerevisiae*, *Hs* *Homo sapiens*, *Dm* *Drosophila melanogaster*, *At* *Arabidopsis thaliana*. **e** The structure of SANT_B with conserved hydrophobic residues shown in stick model.

The preHSA domain of Snf2, consisting of two α helices, adopts a “V” shape (Fig. 4a, b and Supplementary Fig. 9a). The SANT_B domain couples with Snf12_α7 to interact with preHSA_α1 (residues 523–557) by hydrophobic bonds (Fig. 4a). Residues from preHSA_α2 (Asp506, Asp514, and Glu520) form a negatively charged surface to engage positively charged residues from Swp82_α1 (Lys32, Arg36, and Arg40) (Fig. 4c).

Previous studies have indicated that the SANT domains play a central role in chromatin remodeling^29,30^, but the molecular mechanism remains controversial. Sequence alignment of SANT domains from different species showed that the pocket surrounded by SANT_α1-3 contains several highly conserved hydrophobic residues (Trp527, Lue535, Lue536, Trp546, Val553, Cys561, and Phe565) (Fig. 4d, e and Supplementary Fig. 4g) and interacts with the hydrophobic surface of preHSA_α1. The double mutation W546A/F565A and the deletion of the SANT_α3, which disrupted the interaction between the SANT_B and preHSA domains, resulted in a five- to tenfold reduction in the HO-lacZ expression^29^. Importantly, in the structure of the RSC complex, the SANT domain also interacts with the preHSA_α1 of Sth1 in the same way (Supplementary Fig. 9b), although the PS domain of the RSC complex is much larger and more complicated. For BAF complex, the stabilization of preHSA domain of Arg1 mainly depends on the SANT domain of Baf170 (Supplementary Fig. 9c). In summary, these structural features strongly imply that the SANT domains play a conserved and central role in stabilizing the preHSA domain.

### Structural comparison of the base module

**Fig. 5:**
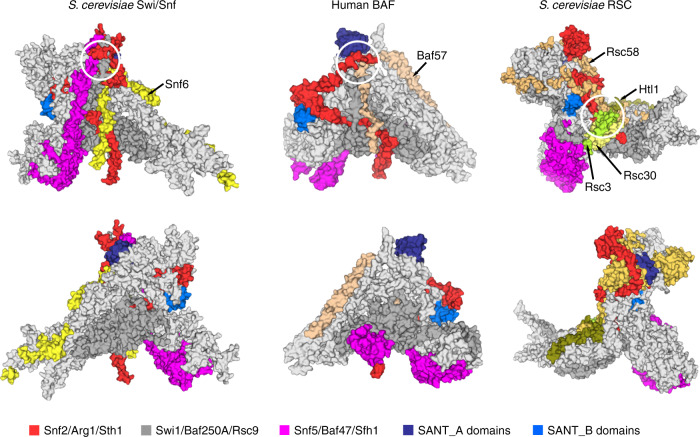
Structural comparison of base modules from Swi/Snf, BAF, and RSC complexes. The front (above) and back (bellow) views are presented in surface model with the multi-subunit junction circled by white lines. The protein elements are color coded and tabulated on the bottom.

The yeast Swi/Snf complex is a homolog of the human BAF complex, which includes multiple paralogs in different tissues and cells, generating extensive diversity in its composition. However, structural comparison of the Swi/Snf complex with the recently published human BAF complex revealed a similar triangular shape. In addition, both complexes have a multi-subunit junction in a similar location; in the BAF complex, Arg1, Baf250A, Baf57, and SANT_A domain of Baf170 merge at a four-way junction, while in the Swi/Snf complex, Snf2, Swi1, Snf6, the SANT_A domain of Swi3, and Snf5 merge at a five-way junction (Fig. 5). Notably, unlike Baf47, it is noteworthy that the N-terminus of Snf5 has two additional α helices that closely hug the rest of the complex closely like a long arm (Fig. 5 and Supplementary Fig. 10a). The α1 helix of Snf5 interacts with the SANT_A domain and α2 forms a coiled coil with Swi1_α1 (Supplementary Fig. 10b, c). This structural feature was not identified in the human Baf47, which may result from the low sequence identity of its N-terminal region with that of Snf5 (Supplementary Fig. 5a) or different experimental conditions.

The RSC complex, another SWI/SNF family remodeler in yeast, differs from the Swi/Snf complex in both component organization and physiological function. Unlike that of the Swi/Snf and BAF complexes, the base module of the RSC complex is divided into three lobes. Its larger PS module (also known as the HB lobe in previous reports), which contains several histone tail-binding bromodomains, splits from the CC domain and Rsc9 to provide flexibility to connect with its substrates.

## Discussion

In this work, we determined the structure of the yeast Swi/Snf complex in a nucleosome-free state. Our structure provides several insights into the overall structural organization of the SWI/SNF remodeler family. In summary, Swi1 and Swi3 form the backbone of the complex and closely connect with surrounding subunits. The hydrophobic pocket of SANT_B stabilizes the preHSA_α1. Surprisingly, the yeast Swi/Snf complex specific protein Snf6 plays a key role in locking Snf2_preHSA on the rigid core. Moreover, Snf5 extends two α helices, further strengthening the complex. These structural characteristics make the base module highly compact, which partially explains why the deletion of Swp82, located in the periphery of the base module (Supplementary Fig. 11), did not result in dissociation of the complex or the loss of other subunits in previous studies^16,17,31^.

By comparing the structures of the yeast Swi/Snf and human BAF complexes, we find that the triangular base module is conserved in this subfamily of SWI/SNF remodelers despite their distinct subunit organization. However, structures of the yeast RSC complex from another subfamily exhibit obvious discrepancies. In particular, the HB lobe of RSC complex is split from the CC domain and Rsc9, which may facilitate the capture of its substrates. This structural diversity may explain functional divergence between the two subfamilies of SWI/SNF remodelers.

While this manuscript was under preparation, the structure of the yeast Swi/Snf complex in the nucleosome-bound state was reported^32^. Compared to this published structure, our much-improved EM map, with local resolution reaching 2.9 Å in the core region (Supplementary Fig. 12a, b), allows unambiguous atomic modeling and identification of key structural features. These newly identified structural features are known to play indispensable roles in maintaining the integrity and stability of the entire complex. For example, the α1-2 of Snf5 and α1 of Swi1 were unassigned in the published structure; however, we find these three α helices cooperate with Snf6 to stabilize Snf2 (Fig. 1c). In addition, by comparing the structures in the nucleosome-free/bound states we find that the base module is well overlapped while the Arp module was rotated approximately 10° away from the NB module to accommodate the nucleosome (Supplementary Fig. 12c, d).This structural rearrangement reveals the dynamic process during nucleosome recognition and binding.

Considering that the *S. cerevisiae* Swi/Snf complex is a classical model, our structure advances mechanistic understanding of complex organization and will shed light on investigation of more complicated SWI/SNF complexes from different species.

## Methods

### *S. cerevisiae* strain

The *S. cerevisiae* strain used to purify Swi/Snf complex carries a 3-Flag tag (DYKDHDGDYKDHDIDYKDDDDK) at the C-terminus of the Snf6 protein. A 3-Flag tag and a HphMX6 marker were amplified by polymerase chain reaction (PCR) from the plasmid pF6Aa-C3FLAG-HphMX6. Using homologous recombination strategy, the PCR products were transformed into a wild-type (trp^−^) *S. cerevisiae* strain (kindly provided by Dr. Rui Bai) by the lithium acetate method. PCR and western blot were used to confirm the correct integration of the 3-Flag tag. Primer sequences are shown in Supplementary Table 2.

### Purification of Swi/Snf complex

Purification of the yeast Swi/Snf complex was modified from a published protocol^6^. The *S. cerevisiae* culture was grown on 1× YPD medium for 8–10 h at 30 °C to an OD600 of ~6. Cell pellets from 36-L culture were collected by centrifugation at 4500 × *g* and washed with buffer containing 1 mM penylmethylsulfonyl fluoride (PMSF). The pellets were resuspended in cold lysis buffer containing 20 mM N-2-hydroxyethylpiperazine-N’-2-ethanesulfonic acid (HEPES) pH 7.4, 350 mM NaCl, 10% glycerol, 0.1% Tween and protease inhibitors (1 mM PMSF, 2.6 μg ml^−1^ aprotinin, 1.4 μg ml^−1^ pepstatin and 5 μg ml^−1^ leupeptin). The cell suspension was dropped into liquid nitrogen to form yeast beads and ground at 18,000 r.p.m. in a Retsch ZM200 nitrogen mill. The yeast powder was thawed at room temperature and centrifuged at 40,000 × *g* for 1 h. The supernatant was loaded into ANTI-FLAG M2 resin (Sigma) and eluted by elution buffer containing 0.5 mg ml^−1^ FLAG peptide (DYKDDDDK), 20 mM HEPES pH 7.4, 350 mM NaCl and protease inhibitors. The complex was concentrated to ~1 mL and applied to a Superose 6 increase 10/300 GL column (GE Healthcare) in SD buffer (20 mM HEPES pH 7.4, 350 mM NaCl). The peak fraction was cross-linked using 1 mM BS3 (Thermo Fisher Scientific) on ice for 2 h, quenched by addition of 50 mM Tris pH 7.4.

### Mass spectrometric analysis

The SDS-PAGE was used to separate the purified Swi/Snf complex and stained with Coomassie Blue G-250. The gel bands of interest were cut into pieces. Sample was digested by trypsin with prior reduction and alkylation in 50 mM ammonium bicarbonate at 37 °C overnight. The digested products were extracted twice with 1% formic acid in 50% acetonitrile aqueous solution and dried to reduce volume by SpeedVac.

For LC-MS/MS analysis, the peptides were separated by a 65-min gradient elution at a flow rate 0.300 µL min^−1^ with the Thermo EASY-nLC1200 integrated nano-HPLC system that is directly interfaced with the Thermo Q Exactive HF-X mass spectrometer. The analytical column was a home-made fused silica capillary column (75 µm ID, 150 mm length; Upchurch, Oak Harbor, WA) packed with C-18 resin (300 A, 3 µm, Varian, Lexington, MA). Mobile phase A consisted of 0.1% formic acid, and mobile phase B consisted of 100% acetonitrile and 0.1% formic acid. The mass spectrometer was operated in the data-dependent acquisition mode using the Xcalibur 4.1 software and there is a single full-scan mass spectrum in the Orbitrap (300–1800 m/z, 60,000 resolution) followed by 20 data-dependent MS/MS scans at 30% normalized collision energy. Each mass spectrum was analyzed using the Thermo Xcalibur Qual Browser and Proteome Discovery for the database searching.

### Cryo-EM specimen preparation and data acquisition

The cross-linked Swi/Snf complex was concentrated to 1 mg mL^−1^ for EM specimen preparation. Briefly, an aliquot of 4-µL sample was applied to a glow-discharged copper Lacey carbon grid (TED PELLA). After waiting for 1 min, the grid was blotted and immersed in uranyl acetate (2%, w/v) for negative staining or rapidly plunged into liquid ethane cooled by liquid nitrogen for cryo-EM specimen. To examine the integrity of the complex, negatively stained sample was imaged on a Thermo Fisher Talos L120C TEM microscope operating at 120 kV. Cryo-EM grids were prepared using Vitrobot Mark IV (FEI Company) operating at 8 °C and 100% humidity.

Cryo-EM specimen were imaged on a 300-kV Titan Krios electron microscope (FEI Company) with a normal magnification of ×81,000. Movies were collected by a Gatan K3 direct electron detector equipped with a GIF Quantum energy filter (slit width 20 eV) at the super-resolution mode. A total 7746 micrographs were automatically recorded using AutoEMation II (developed by Dr. Jianlin Lei)^33^ with a defocus range from −1.5 to −2.0 μm. Each stack of 32 frames were aligned and summed using MotionCor2 ^34^ with a binning factor of 2, resulting in pixel size of 1.087 Å. Dose weighting was performed concurrently^35^. The defocus value for each image was determined by Gctf^36^.

### Cryo-EM image processing

The procedure for image processing of the Swi/Snf complex is outlined in Supplementary Fig. 2. In total, two different batches of particles were automatically picked from 6561 micrographs using Gautomatch (developed by Kai Zhang, https://www.mrc-lmb.cam.ac.uk/kzhang/Gautomatch/), with the second batch particles were auto-picked by excluding the first batch’s bin-1 particles. Reference-free two-dimensional (2D) classification was performed for a small dataset, good classes were selected to generate an initial model using RELION3.0 ^37,38^. Guided multireference classification was applied to the full dataset, details of this modified procedure were previously described^39^. The 3D volumes of the Swi/Snf complex generated from the preliminary data analysis, along with four bad classes, were low-pass filtered to 30 Å and used as the initial references (round 1) (Supplementary Fig. 2). To avoid losing good particles, we simultaneously performed two parallel runs of multireference 3D classification. After round 1, in each batch, good particles from the parallel runs were merged, and the duplicated particles were removed. 444,584 particles and 643,692 particles for the two different batched were selected, respectively (Supplementary Fig. 2).

A second round of multireference local 3D classification (round 2) was performed using the 2× binned particles (pixel size: 2.174 Å). Good classes were merged and duplicated particles were removed to yield 209,583 and 261,977 particles, respectively. Then the two-batch combined 471,560 particles gave rise to a reconstruction of the Swi/Snf complex at an average resolution of 4.1 Å after auto-refinement using unbinned particles (pixel size: 1.087 Å). After that, an additional round (round 3) of 3D classification with local soft-mask was performed. The remaining 386,469 particles yielded a reconstruction at an average resolution of 2.9 Å after ctf-refinement for the main region of the Swi/Snf complex (Supplementary Fig. 2).

All resolutions mentioned above were calculated on the basis of the Fourier shell correlation (FSC) 0.143 criterion^40^, and the FSC curves were corrected with high-resolution noise substitution methods^41^ (Supplementary Fig. 3a). Prior to visualization, all EM maps were corrected for modulation transfer function of the detector, and then sharpened by applying a negative B-factor that was estimated using automated procedures^40^. Local resolution variations were also estimated (Supplementary Fig. 3b). The angular distributions of the particles used for the final reconstruction of Swi/Snf complex are reasonable (Supplementary Fig. 3c), and the refinement of the atomic coordinates did not suffer from severe overfitting (Supplementary Fig. 3d).

### Model building and refinement

The atomic model of the base module from our Swi/Snf complex was mainly built de novo based on our high-resolution map using COOT^42^. De novo model building into the cryo-EM density was guided by the secondary structure prediction of each component from the Swi/Snf complex using the GeneSilico metaserver^43^. In detail, the poly-A sequence was fitted into the map according to helix feature, and bulk residues were used to assign the different components (Supplementary Fig. 4). Part of the Snf12 SWIB domain was homology modeled based on known structure (PDB: 1YCR)^44^. The Arp module was generated from the known structure of the *S. cerevisiae* actin-related subcomplex (PDB: 4I6M)^45^ by docking into our EM density map using UCSF chimera^46^. The position of Arp module was further adjusted and confirmed by overlap region of Snf2 HSA helix (residues 592–605).

The final model of the *S. cerevisiae* Swi/Snf complex was refined according to the high-quality cryo-EM maps using PHENIX in reciprocal space^47^ and secondary structure restraints that were generated meanwhile. Overfitting of the model was monitored by refining the model in one of the two independent maps from the gold-standard refinement approach, and testing the refined model against the other^48^ (Supplementary Fig. 3d). The structure of the Swi/Snf complex was validated through examination of the Molprobity scores and statistics of the Ramachandran plots (Supplementary Table 1). Molprobity scores were calculated as described^49^.

### Reporting summary

Further information on research design is available in the Nature Research Reporting Summary linked to this article.

## Supplementary information

Supplementary information

Reporting summary

## Data Availability

The atomic coordinates have been deposited in the Protein Data Bank with the accession code 7C4J, and the EM maps have been deposited in EMDB with the accession codes EMD-30285 and EMD-30286, respectively. Other data are available from the corresponding authors upon reasonable request.
