# Author Correction: Structure of the yeast Swi/Snf complex in a nucleosome free state

**DOI:** 10.1038/s41467-020-19707-8

**Published:** 2020-11-06

**Authors:** Chengcheng Wang, Zhouyan Guo, Xiechao Zhan, Fenghua Yang, Mingxuan Wu, Xiaofeng Zhang

**Affiliations:** 1 Key Laboratory of Structural Biology of Zhejiang Province, Institute of Biology, Westlake Institute for Advanced Study, 18 Shilongshan Road, 310024 Hangzhou, Zhejiang Province China; 2 School of Life Sciences, Westlake University, 18 Shilongshan Road, 310024 Hangzhou, Zhejiang Province China; 3 College of Life Sciences, Zhejiang University, 310058 Hangzhou, China; 4 Beijing Advanced Innovation Center for Structural Biology, Tsinghua-Peking Joint Center for Life Sciences, School of Life Sciences, Tsinghua University, 100084 Beijing, China; 5 School of Science, Westlake University, 18 Shilongshan Road, 310024 Hangzhou, Zhejiang Province China; 6 Institute of Natural Sciences, Westlake Institute for Advanced Study, 18 Shilongshan Road, 310024 Hangzhou, Zhejiang Province China

**Keywords:** Biochemistry, Cryoelectron microscopy, Molecular biology

Correction to: *Nature Communications* 10.1038/s41467-020-17229-x, published online 7 July 2020

The original version of this Article contained an error in Fig. 3c, where residue Snf6_F103 was mislabelled as Snf6_F102. The Article also contained an error describing Fig. 3c in the “Results” section, which incorrectly referred to Phe80 instead of Phe103. The correct text should read ‘Arg84, the N-terminal residue of Snf6_loop 1, interacts with Asp578 from the HSA helix through hydrogen bonds (Fig. 3b), while the C-terminal residue Phe103 forms Π stacking with the residue Pro565 from Snf2 (Fig. 3c).’

These errors have been corrected in both the PDF and HTML versions of the Article.

