# Supplementary material for: Structure of the yeast Swi/Snf complex in a nucleosome free state
Source: Nat Commun. 2020 Jul 7;11:3398. doi: 10.1038/s41467-020-17229-x (PMC7340788; doi:10.1038/s41467-020-17229-x)
Supplement: Supplementary file 1 — Supplementary information [file 41467_2020_17229_MOESM1_ESM.pdf]

# Supplementary Information

Structure of the yeast Swi/Snf complex in a nucleosome free state

Wang et al.

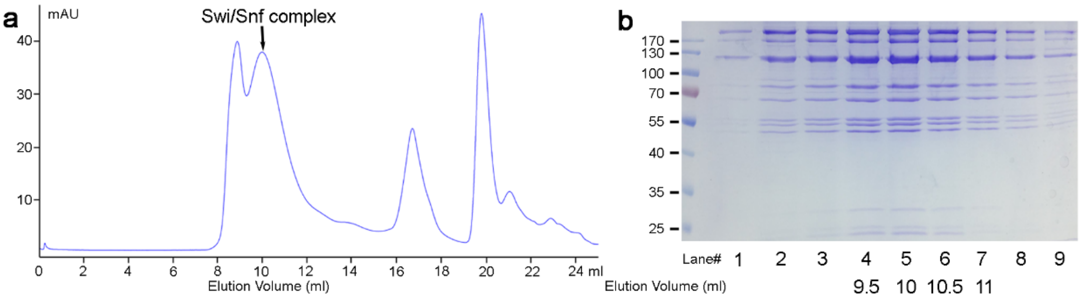


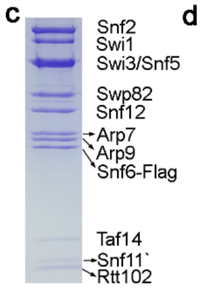


d

| Protein name (*S. cerevisiae*) | Uniport ACC. NO. | #AAs | MW (kDa) | #PSMs | Docked |
|---|---|---|---|---|---|
| Snf2 | P22082 | 1703 | 193.9 | 396 | yes |
| Swi3 | P32591 | 825 | 92.9 | 271 | yes |
| Swi1 | P09547 | 1314 | 147.8 | 214 | yes |
| Arp7 | Q12406 | 477 | 53.8 | 163 | yes |
| Snf12 | P53628 | 566 | 63.8 | 134 | yes |
| Swp82 | P43554 | 623 | 70.2 | 174 | yes |
| Snf5 | P18480 | 905 | 102.5 | 112 | yes |
| Arp9 | Q05123 | 467 | 53 | 102 | yes |
| Snf6 | P18888 | 332 | 37.6 | 29 | yes |
| Taf14 | P35189 | 244 | 27.4 | 20 | no |
| Rtt102 | P53330 | 157 | 17.8 | 17 | yes |
| Snf11 | P38956 | 169 | 18.7 | 7 | no |

e

| | Yeast Swi/Snf | Yeast RSC | Human BAF |
|---|---|---|---|
| Conserved | Snf2 | Sth1 | Brg1/Brm |
| | Swi1 | Rsc9 | Baf250A/B |
| | Swi3 | Rsc8 | Baf155 |
| | | | Baf170 |
| | Snf5 | Sfh1 | Baf47 |
| | Snf12 | Rsc6 | Baf60A/B/C |
| | Arp7 | Arp7 | β-actin |
| | Arp9 | Arp9 | Baf53A/B |
| | Rtt102 | Rtt102 | |
| Specific | Snf6, Swp82, Snf11, Taf14 | Rsc1/2/3/4/ 7/14/30/58, Htl1 | Baf57, Brd9, Baf45, Bcl7, Bcl11, SS18, Gltscr1 |

**Supplementary Figure 1. Purification and characterization of the Swi/Snf complex from *S. cerevisiae*.** (**a**) Gel filtration analysis of the Swi/Snf complex purified by affinity chromatography. (**b**) The peak fractions from gel filtration were visualized on SDS-PAGE by Coomassie blue staining. Fractions 4-7 were collected for cryo-EM sample preparation. (**c**) and (**d**) Protein components of purified Swi/Snf complexes were separated on 12% SDS-PAGE and further confirmed by mass spectrometry analysis. (**e**) Subunit organization of the yeast Swi/Snf, yeast RSC and human BAF complexes.

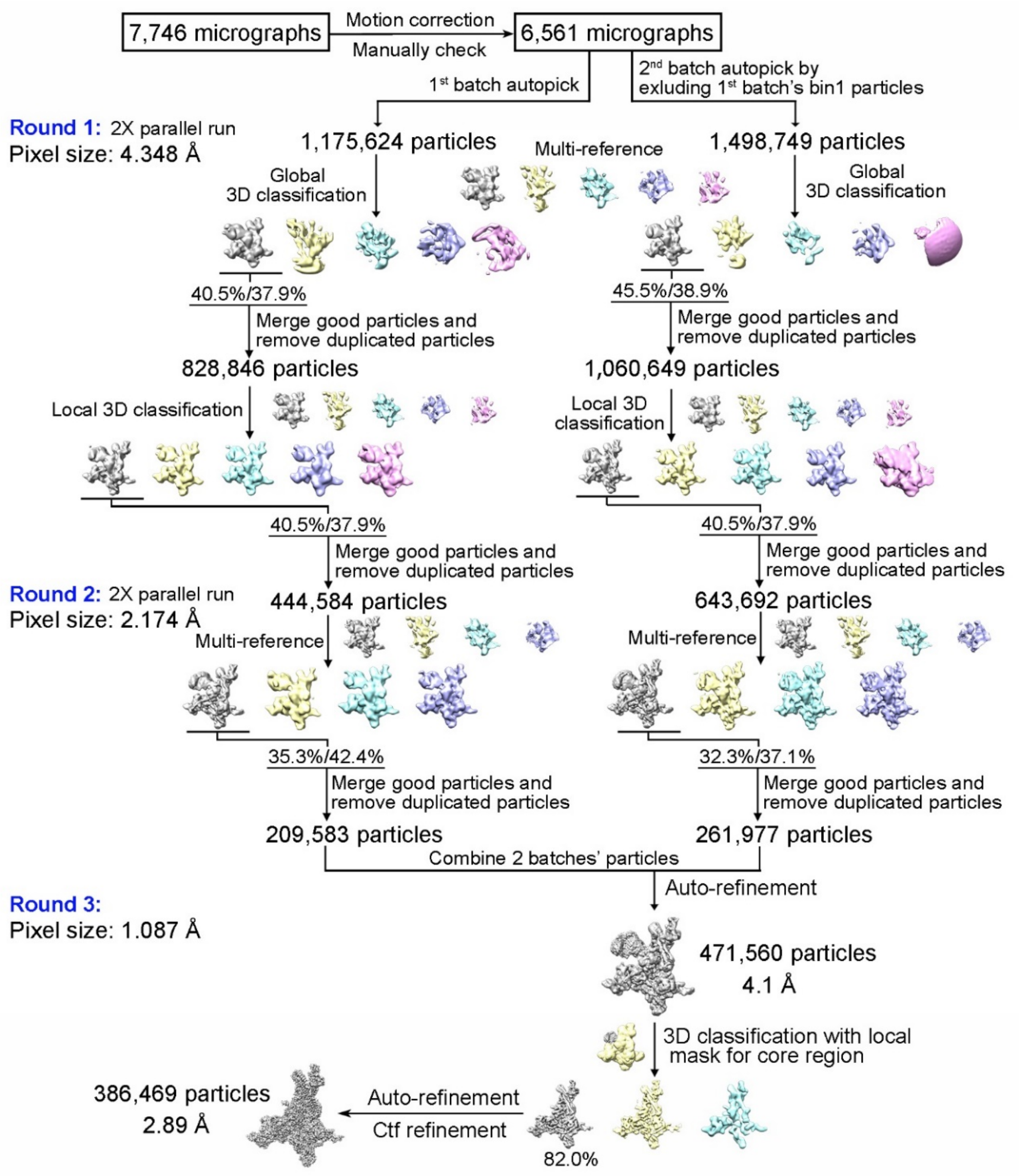


**Supplementary Figure 2. A flow chart description of the EM data processing and structure determination of the *S. cerevisiae* Swi/Snf complex.** The final reconstruction has an average resolution of 4.1 Å for the Swi/Snf complex on the basis of the FSC value of 0.143. Please refer to Methods for details.

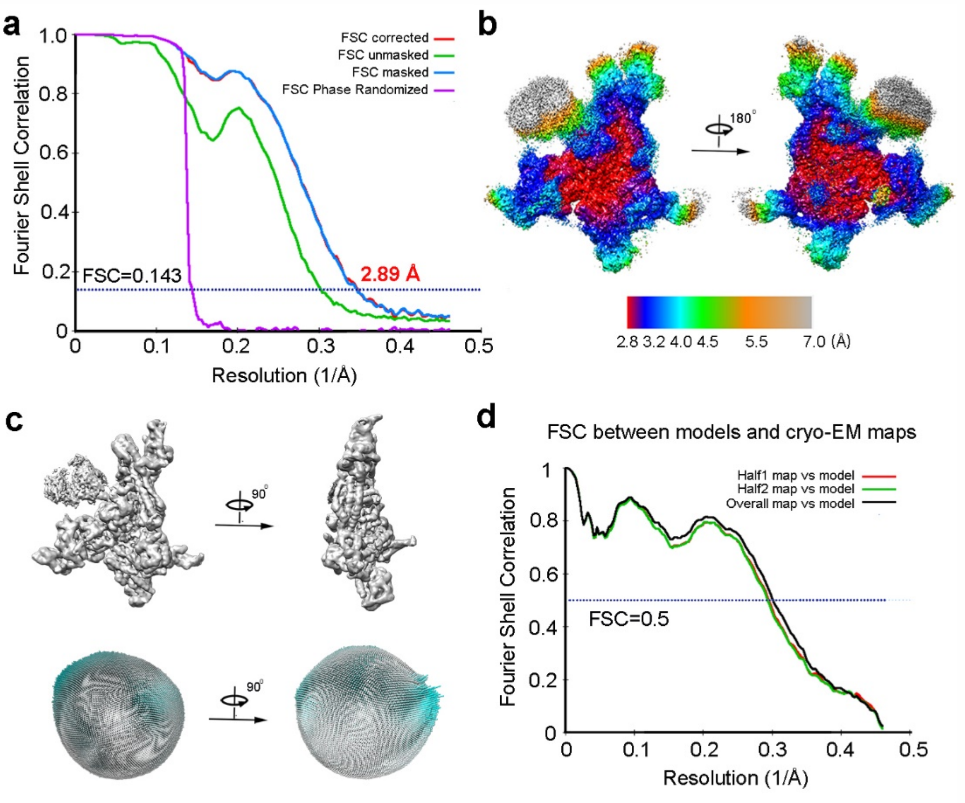


**Supplementary Figure 3. Cryo-EM analysis of the *S. cerevisiae* Swi/Snf complex.** (**a**) The average resolution is estimated to be 2.89 Å for the reconstruction of the Swi/Snf complex main region on the basis of the FSC criterion of 0.143. (**b**) The local resolutions are color-coded for different regions of the Swi/Snf complex. (**c**) Angular distribution of the particles used for the reconstruction of the Swi/Snf complex. Each cylinder represents one view and the height of the cylinder is proportional to the number of particles for that view. (**d**) The FSC curves of the final refined model versus the overall map it was refined against (black); of the model refined in the first of the two independent maps used for the FSC calculation versus that same map (red); and of the model refined in the first of the two independent maps versus the second independent map (green). The generally similar appearances between the red and green curves indicates that the refinement of the atomic coordinates did not suffer from severe over-fitting.

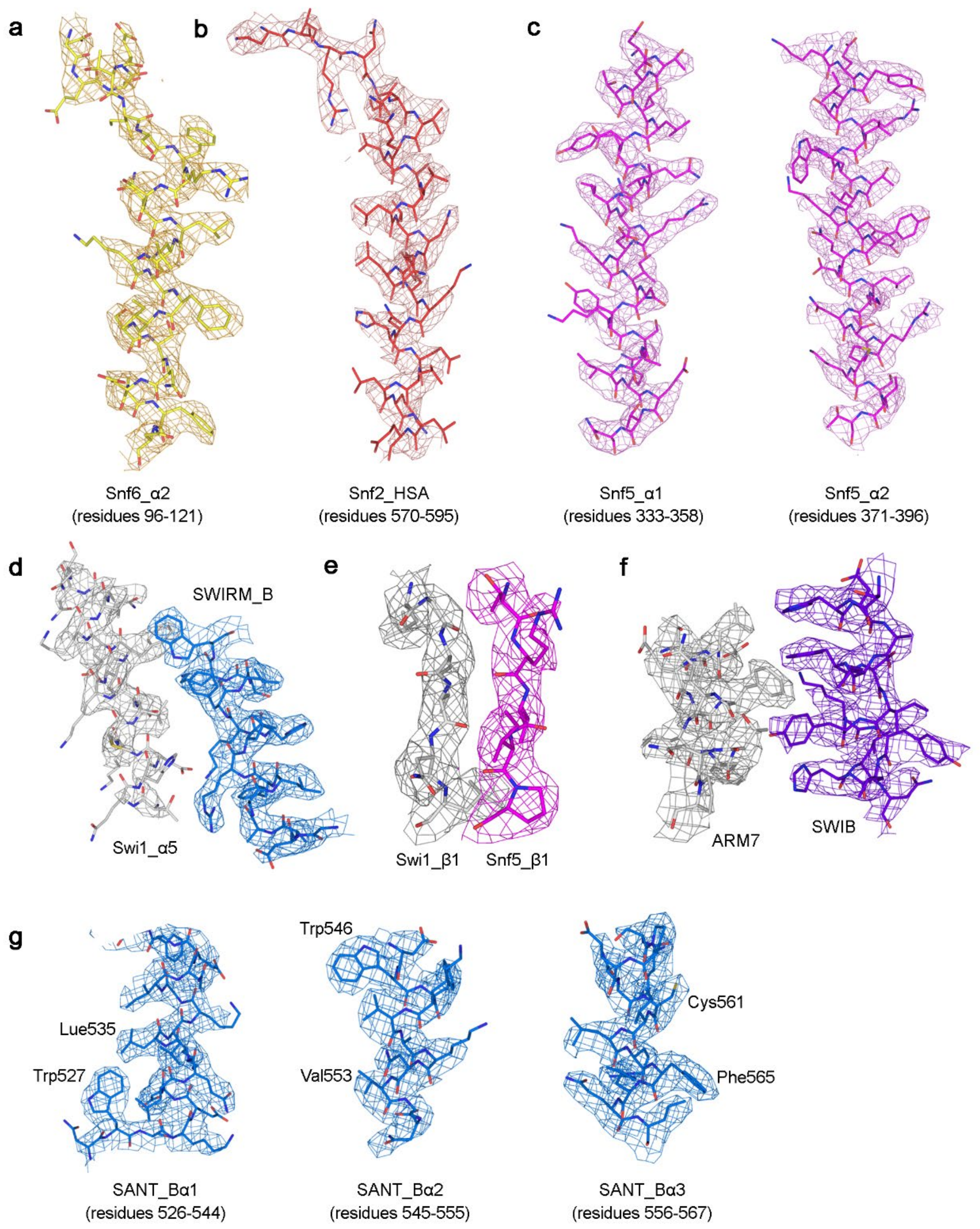


**Supplementary Figure 4. EM maps for representative segments of the Swi/Snf complex.** (**a**) The EM map for the Sn6_α2. (**b**) The EM map for the Sn2_HSA. (**c**) The EM map for the Sn5_α1 and α2. (**d**) The EM map of Swi1_α5 and SWIRM_B domain. (**e**) The EM map for the Swi1_β1 and Sn5_β1. (**f**) Local EM map for the ARM7 and SWIB. (**g**) The EM maps for the α1-3 of the SANT_B domain.

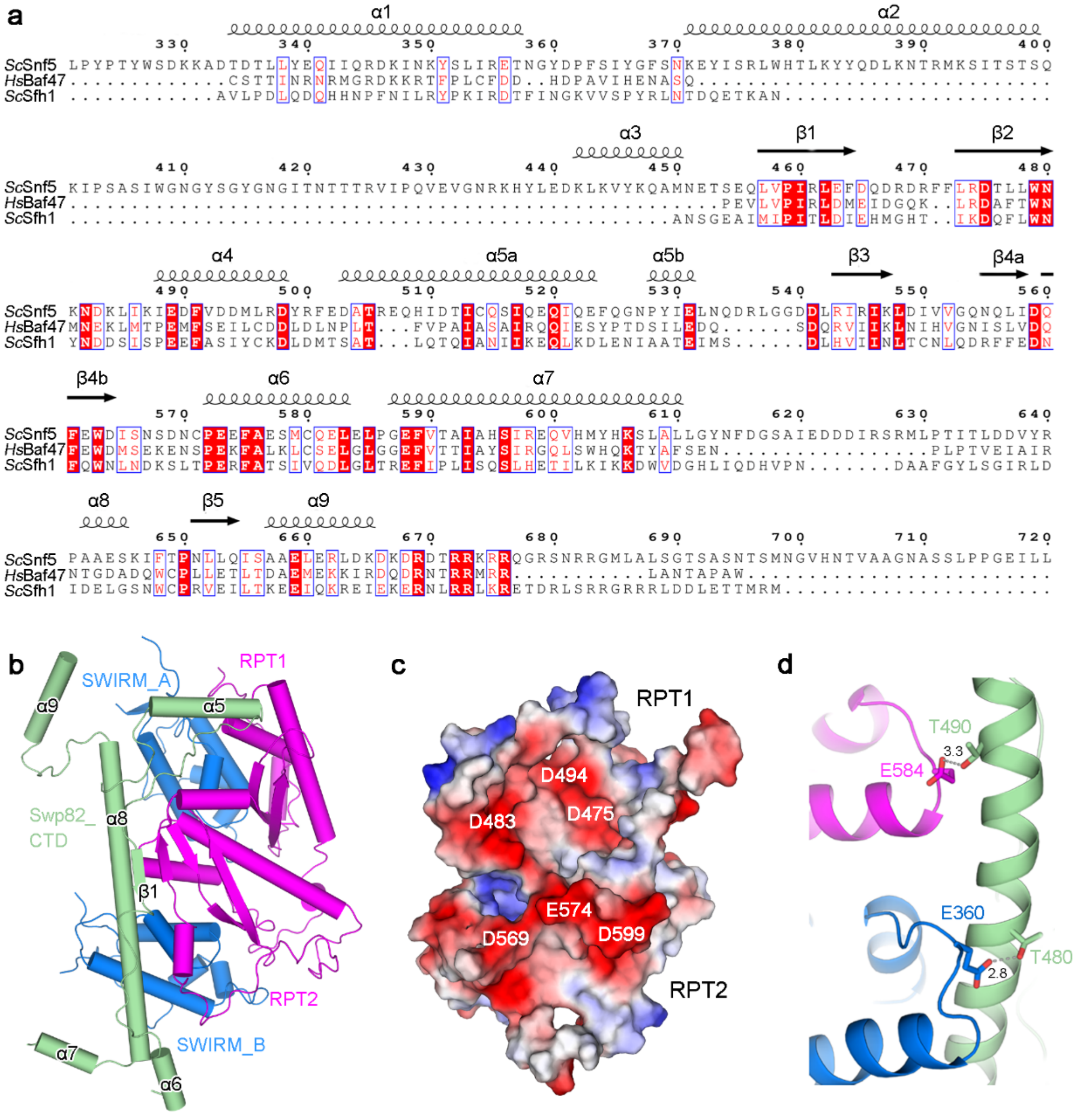


**Supplementary Figure 5. Structure of the NB module.** (**a**) Sequence alignment of *Sc*SnF5, *Hs*Baf47 and *Sc*Sfh1. *Sc*, *Saccharomyces cerevisiae*; *Hs*, *Homo sapiens*. (**b**) Cylinder schematic of NB module. (**c**) Electrostatic potential map of RPT domains of Snf5. The negative-charged residues interacting with SWIRM domains are labeled on the map. (**d**) Swp82_CTD stitches RPT and SWIRM domains together.

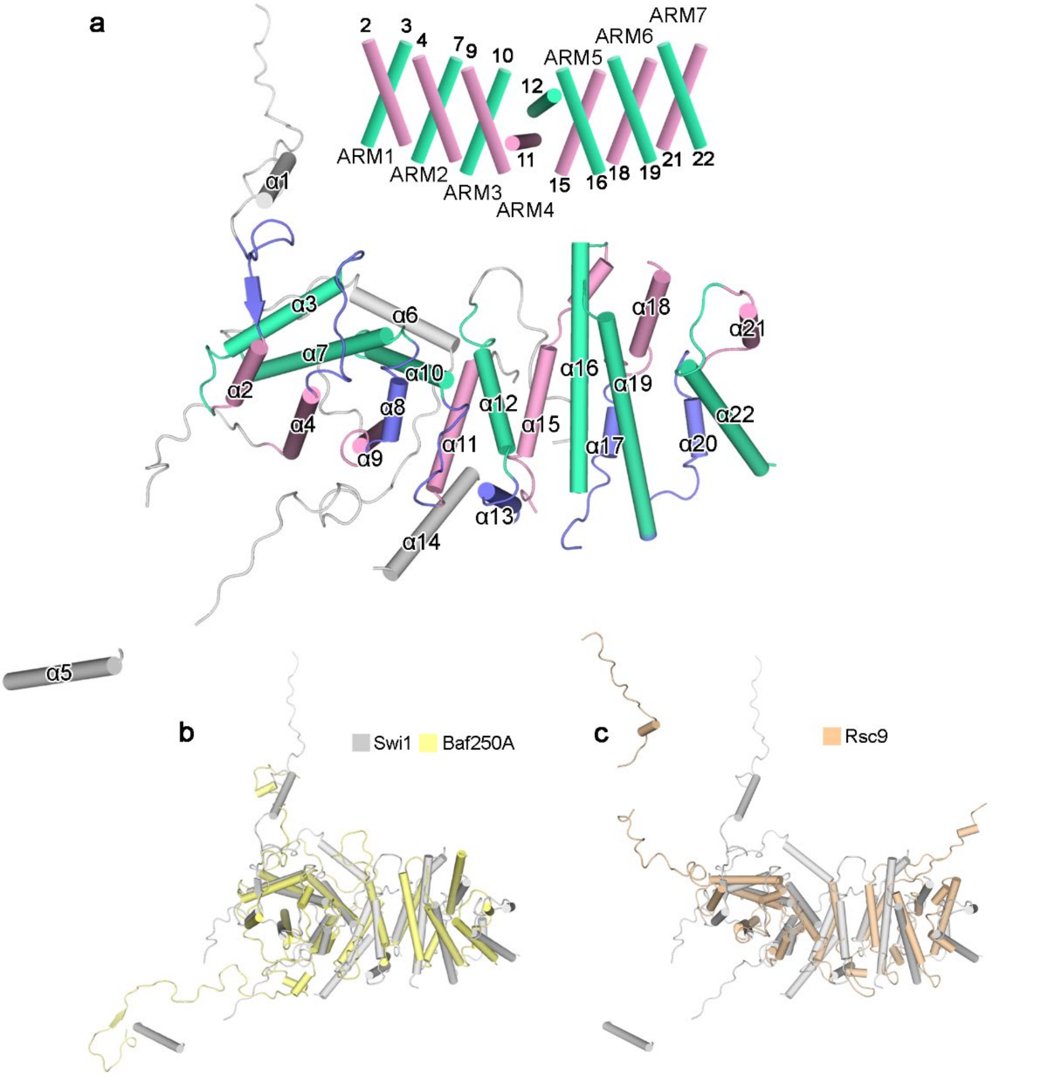


**Supplementary Figure 6. Structure of the C-terminal domain of Swi3.** (**a**) Topology of ARM repeats and the structure of C-terminus of Swi1. (**b**) and (**c**) Comparison between Swi1 with Baf250A (PDB: 6LTH) or Rsc9 (PDB: 6K15).

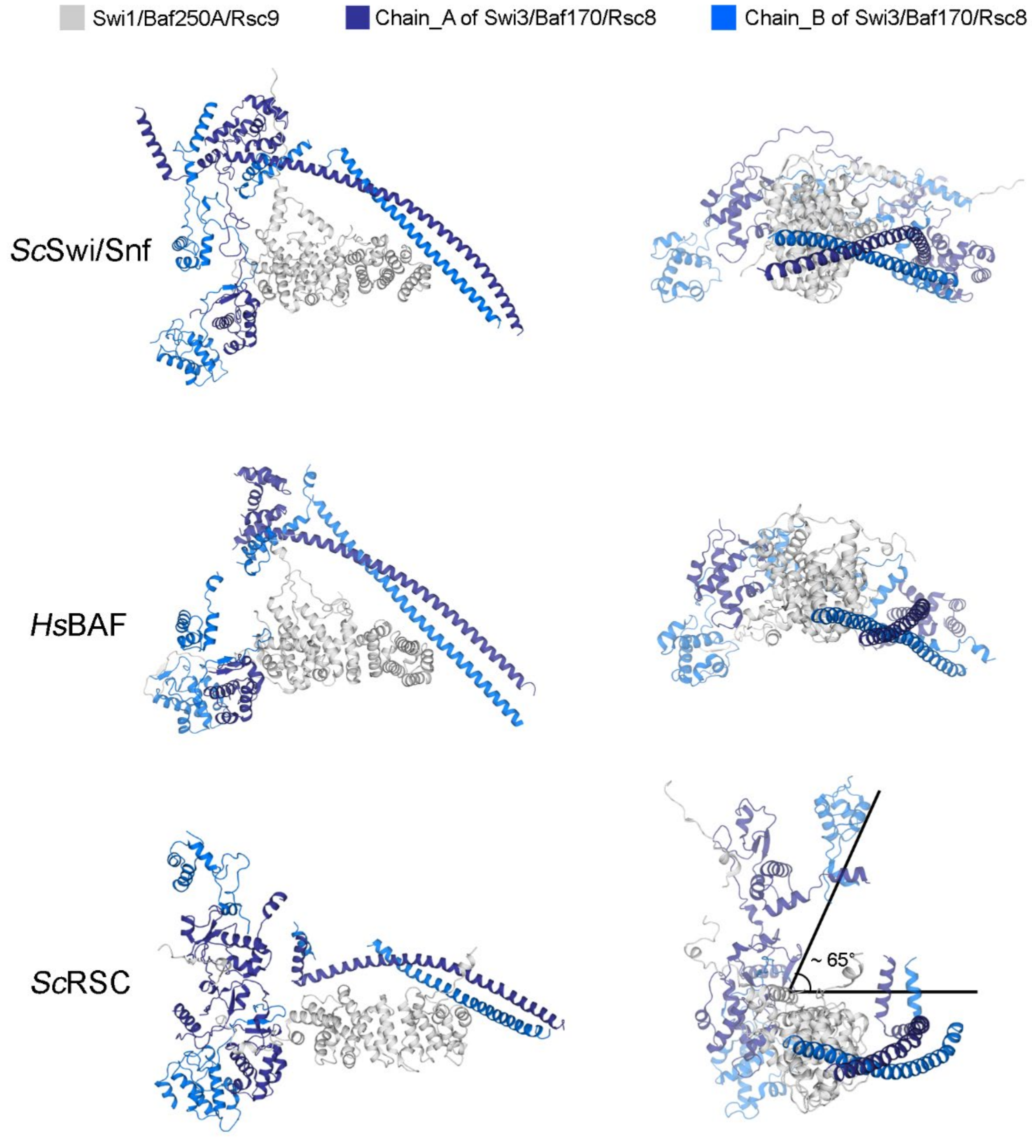


**Supplementary Figure 7. The backbone of yeast Swi/Snf complex formed by Swi1_Swi3 and comparison with human BAF complex (PDB: 6LTH) and yeast RSC complex (PDB: 6K15).** Two different views are presented. The proteins are color coded and tabulated on the top.

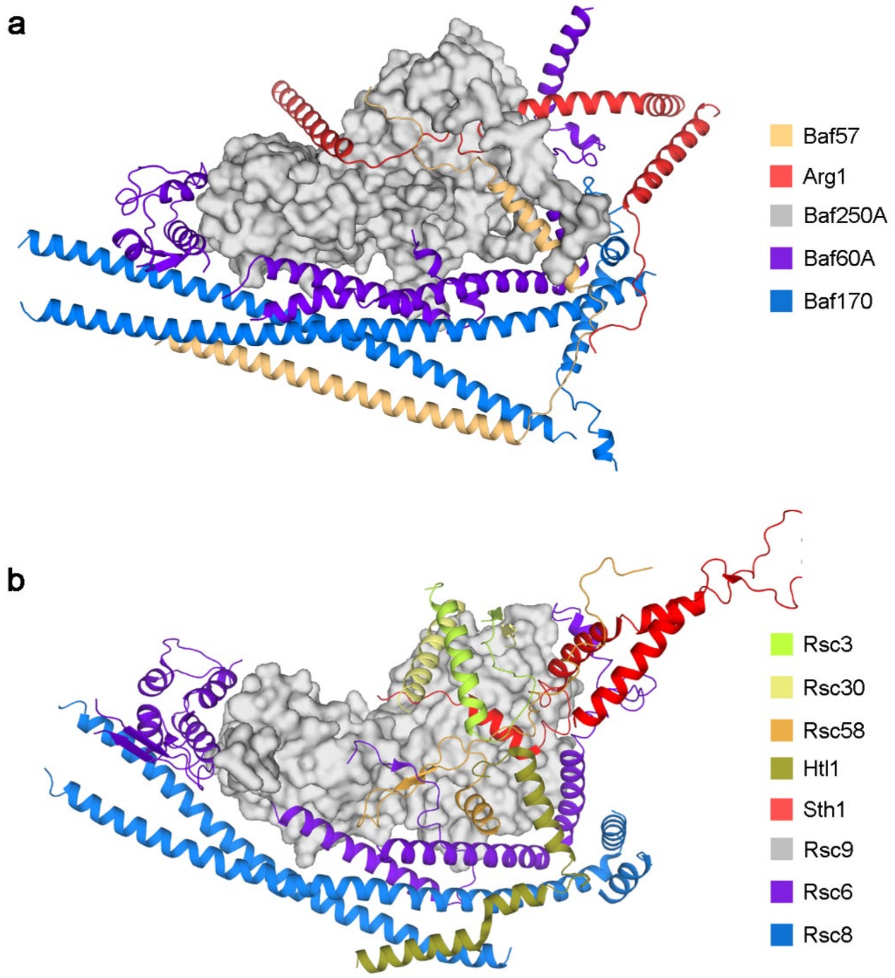


**Supplementary Figure 8**. **Stabilization of preHSA loop in the structure of human BAF complex (PDB: 6LTH) and yeast RSC complex (PDB: 6K15).** **(a)** Baf57 locks the preHSA loop of Arg1 on the surface of Baf250A in a similar manner with yeast Snf6. **(b)** Rsc3, Rsc30, Rsc58 and Htl1 collaboratively anchor the preHSA loop of Sth1 on the surface of Rsc9. The protein elements are color coded and tabulated on the right.

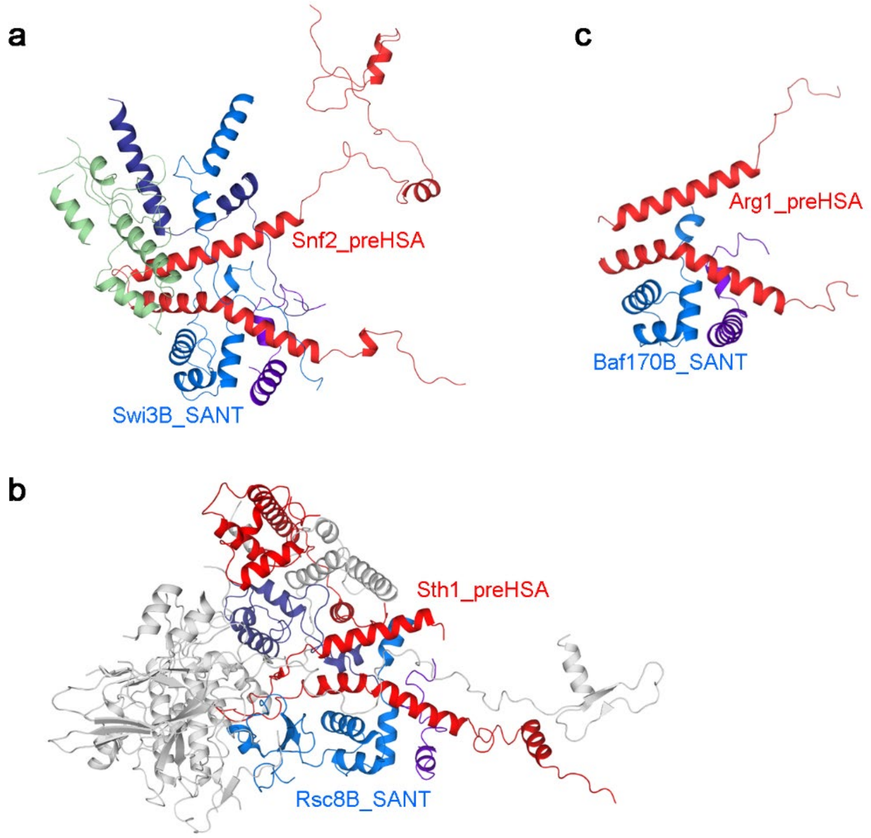


**Supplementary Figure 9. Structural comparison of PS modules from yeast Swi/Snf complex (a), RSC complex (b) and human BAF complex (c).**

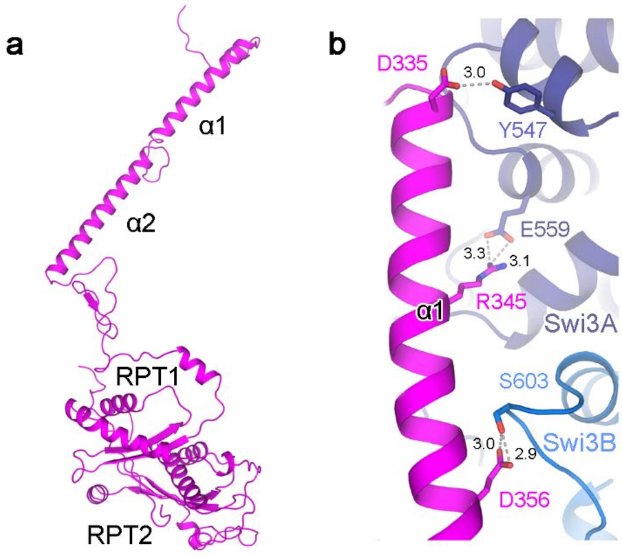


**Supplementary Figure 10. Structure of Snf5.** (**a**) Overall organization of Snf5 shown in carton representation. (**b**) Interactions between Snf5_α1 and Swi3_A/B. Key residues are labelled and shown in stick mode. (**c**) Snf5_α2 forms coiled coil with Swi1_α1.

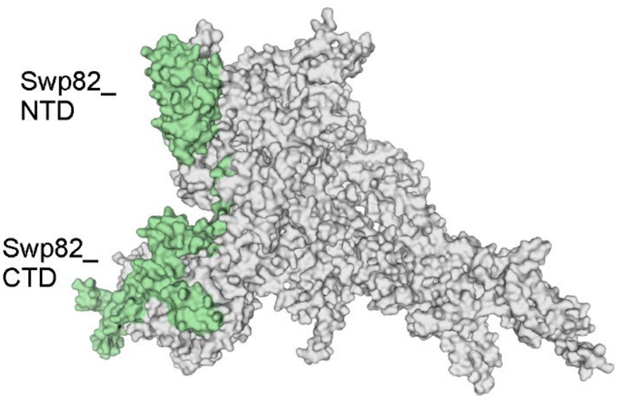


**Supplementary Figure 11. Swp82 is located in the peripheral of the base module. The base module is shown in surface representation with Swp82 colored green.**

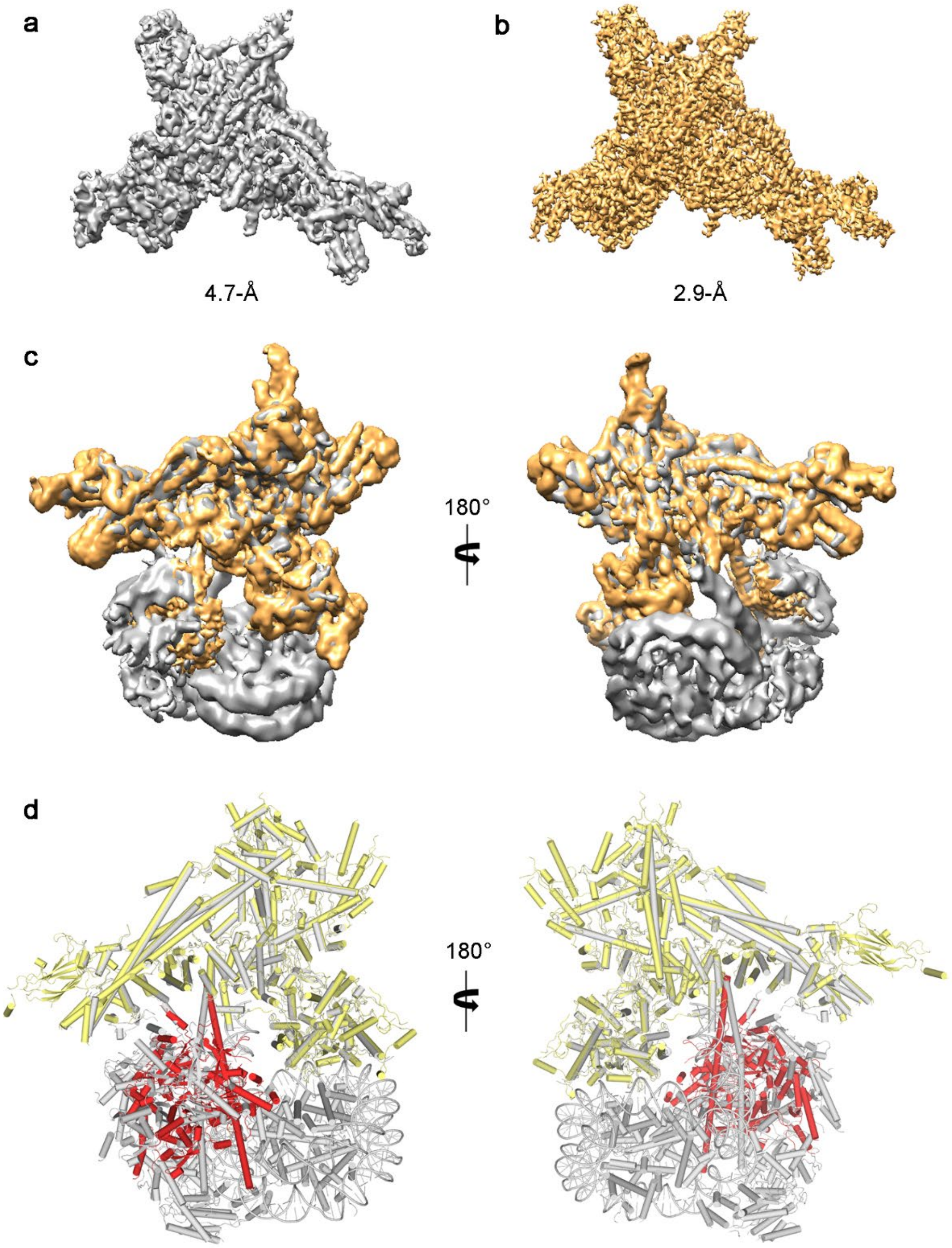


**Supplementary Figure 12. Structural comparison of the yeast Swi/Snf complex in the nucleosome-bound state and nucleosome-free state.** (**a**) The EM map (EMD_20933) for the base module by *Han et al.* is colored in grey. (**b**) The EM map for the base module at a resolution of 2.9 Å is colored in orange. (**c**) and (**d**) Comparison of the yeast Swi/Snf complex in the nucleosome-bound state and nucleosome-free state. The EM map (EMD_20934) and the cartoon model (PDB: 6UXW) in the nucleosome-bound state is colored in grey. The EM map in the nucleosome-free state is colored in orange (**c**). The base module and Arp module in the nucleosome-

free state are shown by cartoon model and colored in yellow and red, respectively (**d**).

**Supplementary Table 1. Cryo-EM data collection and refinement statistics.**

| | |
|---|---|
| **Data collection** | |
| EM equipment | FEI Titan Krios |
| Voltage (kV) | 300 |
| Detector | Gatan K3 |
| Pixel size (Å) | 1.087 |
| Electron dose (e-/Å$^2$) | 50 |
| Defocus range (μm) | 1.2~2.5 |
| **Reconstruction** | |
| Software | RELION-3.0 |
| Number of used Particles | 386,469 |
| Accuracy of rotation (˚) | 0.47 |
| Accuracy of translation (Å) | 0.55 |
| Final Resolution (Å) | 2.89 |
| **Model building** | |
| Software | COOT |
| **Refinement** | |
| Software | PHENIX |
| Map sharpening B-factor (Å$^2$) | -69. 97 |
| Average Fourier shell correlation | 0.69 |
| R-factor | 0.43 |
| **Model composition** | |
| Protein residues | 3717 |
| **Validation** | |
| R.m.s deviations | |
| Bonds length (Å) | 0.007 |
| Bonds Angle (˚) | 0.914 |
| Ramachandran plot statistics (%) | |
| Preferred | 90.82 |
| Allowed | 6.05 |
| Outlier | 3.66 |
| Molprobity score | 2.76 |

**Supplementary Table 2. Primer sequence used in this study.**

| | Sequence (5'→3') (HA: homologous arm) |
|---|---|
| Snf6-HA1-F | AGGGATGTTTTGGCGCAATA |
| Snf6-HA1-R | AAAAAATACAGCATCAAGATCTCC |
| Snf6-HA2-F | TAGCCATGGGCGGCTACT |
| Snf6-HA2-R | GTTTACGACTGGCACATCTCG |
| C3FLAG-HphMX6-F | TGGAGATCTTGATGCTGTATTTTTTCGGATCCCCGGGTTAATT |
| C3FLAG-HphMX6-R | GAAGTAGCCGCCCATGGCTAGAATTCGAGCTCGTTTAAAC |
